# Rapid Temporal Recalibration to Audiovisual Asynchrony Occurs Across the Difference in Neural Processing Speed Based on Spatial Frequency

**DOI:** 10.1177/2041669520966614

**Published:** 2020-10-30

**Authors:** Yasuhiro Takeshima

**Affiliations:** Department of Psychology, Doshisha University, Kyoto, Japan

**Keywords:** multisensory/cross-modal processing, temporal processing, visuoauditory interactions, synchrony

## Abstract

Audiovisual integration relies on temporal synchrony between visual and auditory stimuli. The brain rapidly adapts to audiovisual asynchronous events by shifting the timing of subjective synchrony in the direction of the leading modality of the most recent event, a process called rapid temporal recalibration. This phenomenon is the flexible function of audiovisual synchrony perception. Previous studies found that neural processing speed based on spatial frequency (SF) affects the timing of subjective synchrony. This study examined the effects of SF on the rapid temporal recalibration process by discriminating whether the presentation of the visual and auditory stimuli was simultaneous. I compared the magnitudes of the recalibration effect between low and high SF visual stimuli using two techniques. First, I randomly presented each SF accompanied by a tone during one session, then in a second experiment, only a single SF was paired with the tone throughout the one session. The results indicated that rapid recalibration occurred regardless of difference in presented SF between preceding and test trials. The recalibration magnitude did not significantly differ between the SF conditions. These findings confirm that intersensory temporal process is important to produce rapid recalibration and suggest that rapid recalibration can be induced by the simultaneity judgment criterion changes attributed to the low-level temporal information of audiovisual events.

Multisensory integration is an important function in human sensory information processing. In audiovisual integration, auditory stimuli increase the salience of visual stimulus (e.g., Noesselt et al., 2008; Stein et al., 1996). Many studies have reported that temporal synchrony between visual and auditory stimuli is necessary for audiovisual integration. For example, visual target detectability can be improved by temporally consistent auditory stimulus (Bolognini et al., 2005). Furthermore, simultaneous auditory stimulus increases the accuracy of visual target in the rapid serial visual presentation task (Olivers & van der Burg, 2008).

The synchrony perception for audiovisual stimuli, based on audiovisual integration, must to accommodate the lags between visual and auditory stimuli. First, there are differences in the transmission times of light and sound, and second, the neural response latencies are different between visual and auditory sensations (King, 2005). Thus, the point of subjective simultaneity (PSS) often differs from physical synchrony timing (for a review, see Vroomen & Keetels, 2010). Moreover, a perfect temporal alignment is not a prerequisite to perceive audiovisual synchrony such as sounds of 100 to 200 ms that precede and follow visual stimuli (Dixon & Spitz, 1980; Guski & Troje, 2003), this range is often referred to the temporal binding window. In addition, our brains realign audio-visual signals, as shown in a number of studies demonstrating that repeated exposure (lasting up several minutes) to audiovisual asynchrony shifts the PSS in the direction of the leading sense; this adaptive phenomenon is known as temporal recalibration (e.g., Fujisaki et al., 2004; Vroomen et al., 2004). On the other hand, van der Burg et al. (2013) reported temporal recalibration without a crossmodal adaptation procedure, which they termed “rapid recalibration (van der Burg et al., 2013, p. 14633).” In rapid recalibration, the PSS in a trial is contingent upon the audiovisual asynchrony in the preceding trial. The rapid recalibration process has large transient effects compared with a typical recalibration with a crossmodal adaptation procedure (van der Burg et al., 2015).

Rapid recalibration is proposed to depend on only temporal information (i.e., stimulus onset asynchronies: SOAs) of preceding audiovisual stimuli. Harvey et al. (2014) have reported that rapid recalibration occurs even if visual (i.e., colors or orientations) or auditory (i.e., frequency) features of the preceding (adaptation) trial differs from the current (test) trial. In using audiovisual speech stimuli pronounced by an actor, rapid recalibration occurs even when auditory and visual events clearly belong to different actors in the preceding trial (van der Burg & Goodbourn, 2015). In addition, rapid recalibration can be observed regardless of the spatial location of audiovisual stimuli between preceding and current trials (Ju et al., 2019). Van der Burg et al. (2013, 2018) have shown that the PSS is shifted based on the physical timing of the preceding audiovisual stimuli, not the perceived timing. Thus, the physical temporal information of an audiovisual event is important for producing rapid recalibration (Harvey et al., 2014).

However, the effects of neural processing speed on rapid temporal recalibration have not yet been elucidated. Although, previous studies have not proposed that various sensory features (e.g., color, orientation, or location) affect rapid recalibration as mentioned earlier (Harvey et al., 2014; Ju et al., 2019), these studies have not manipulated the sensory features related to temporal processing. It is possible that the difference of processing speed between preceding and/or current trials play a role in rapid recalibration. Neural response latencies differed between different spatial frequencies (SFs) of visual stimuli, which are processed in the lateral geniculate nucleus and primary visual cortex (Breitmeyer, 1975). Low SFs are preferably processed in transient channels, which are characterized by fast response onset. On the other hand, high SFs are preferably processed in sustained channels, which are characterized by slow response onset. Furthermore, PSS scores are modulated by the SF of visual stimuli (Takeshima & Gyoba, 2015; Tappe et al., 1994). Compared with high SFs, low SFs shift PSS in the direction of auditory precedence.

Therefore, this study examined whether the difference of processing speed with SF of visual stimuli modulates rapid temporal recalibration using a simultaneity judgment (SJ) task. The difference in SF of visual stimuli alter the neural processing speed (Breitmeyer, 1975) and also the PSS for audiovisual stimuli (Takeshima & Gyoba, 2015; Tappe et al., 1994). This study investigated whether the magnitude of rapid recalibration is modulated not only by the physical timing of the preceding trial but also by neural processing speed in the preceding and current trials. In addition, the simple reaction time (RT) measurement for low and high SF visual stimuli was conducted to replicate Breitmeyer (1975). The PSS of low SF visual stimuli is biased to auditory precedence compared with high SF visual stimuli (Takeshima & Gyoba, 2015; Tappe et al., 1994). Therefore, auditory leading is expected to affect less the low than the high SF visual stimuli, thereby reducing the rapid recalibration magnitude for the low SF visual stimuli. This research could help elucidate the temporal information needed for rapid recalibration to occur.

## Experiment 1

In Experiment 1, I examined whether the magnitude of rapid recalibration could be modulated by the difference in SF of visual stimuli presented in preceding or current trials, using a SJ task. Two different Gabor patches displaying different SFs were randomly presented with an auditory beep by one of the 10 SOAs in this experiment. The magnitudes of rapid recalibration were compared between SFs in the current trial. Moreover, the effects of difference in SFs in the preceding trial were investigated.

### Method

#### Participants

A total of 33 individuals (26 women and 7 men; mean age = 19.18 ± 1.57 years) participated in this experiment. All participants orally reported normal or corrected-to-normal vision and normal hearing and provided written informed consent before participation. This study was approved by the ethics committee of Doshisha University (no. 17094).

#### Apparatus

Stimuli were generated and controlled by means of a custom-made program written in MATLAB (The MathWorks, Inc.), Psychtoolbox (Brainard, 1997; Kleiner et al., 2007; Pelli, 1997), and a laptop PC (MacBook Pro, Apple, Cupertino/USA). The visual stimuli were displayed on a 21-inch cathode-ray tube display (Trinitron CPD-G520, Sony; resolution: 1,024 × 768 pixels; refresh rate: 100 Hz). The auditory stimuli were conveyed through an audio interface (Clarett 2Pre, Focusrite, High Wycombe/England) and headphones (MDR-CD900ST, Sony, Minato-ku/Japan). The simultaneity of the visual and auditory stimuli was confirmed using a digital oscilloscope (DS-5424A, Iwatsu, Suginami-ku/Japan). The experiment was conducted in a dimly darkened room with 39.8 dB (A) of background noise. Participants viewed the monitor binocularly at a distance of 70 cm with their heads stabilized on a chin rest.

#### Stimuli

**Figure 1.**
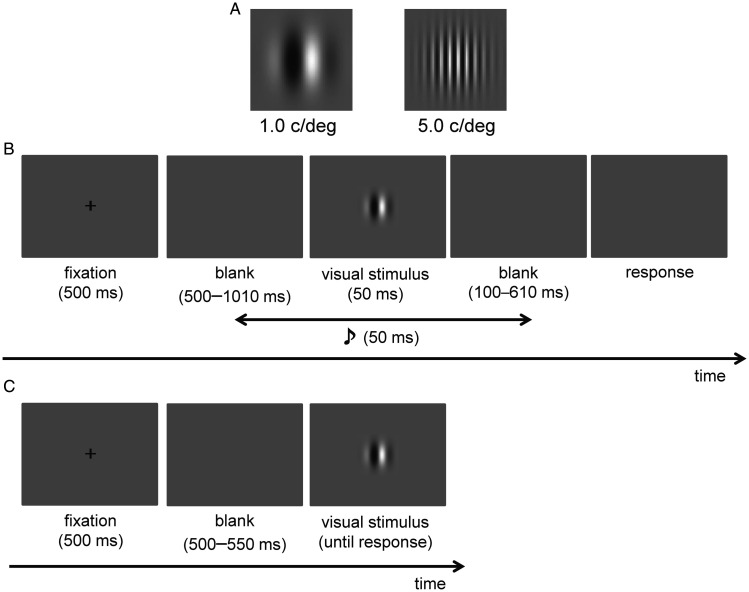
A: Low spatial frequency (1.0 c/deg; left) and high spatial frequency (5.0 c/deg; right) Gabor patches used in the experiment. B: Schematic representation of the simultaneity judgment task. C: Schematic representation of the response time experiment.

Visual stimuli were Gabor patches (Figure 1A) of two SFs, 1.0 and 5.0 cycles per degree (c/deg). The size of the visual stimulus was approximately 2.0° in diameter, and the luminance was 3.29 to 122.02 cd/m^2^ with a duration of 50 ms. The fixation point was a white cross (159.51 cd/m^2^) that was 1.1° in diameter. These stimuli were presented on a gray background (29.37 cd/m^2^). The auditory stimulus was a pure tone of 500 Hz with a duration of 50 ms (including ramp times of 5 ms at the beginning and end of the sound wave envelope), and the sound pressure level was 55 dB (A). There were 10 SOAs between visual and auditory stimuli: ±510, ±260, ±130, ±50, and ±0 ms (negative SOAs indicate that the auditory stimulus was presented before the visual stimulus and vice versa).

#### Procedure

A trial schematic is displayed in Figure 1B. Trials were initiated by pressing the 0 on the keyboard. Each trial consisted of a 500-ms fixation followed by blank and visual stimulus presentations. The duration of the blank displays was randomized (500–1010 ms). Visual stimulus was presented for 50 ms accompanying an auditory stimulus randomly selected from the range of SOAs. After presenting the visual stimulus, participants were instructed to judge whether the presentation of the visual and auditory stimuli was simultaneous by pressing 1 for simultaneity and 3 for asynchrony. This SJ task consisted of two sessions, each comprising three blocks. One block consisted of 100 trials and there were 10 trials for each SOA condition. Half the trials of one block had the 1.0 c/deg Gabor patch presented, while the remaining half had the 5.0 c/deg Gabor patch presented. Therefore, each participant completed a total of 600 trials. After completing the SJ task, each participant performed a simple RT task for both Gabor patches to confirm the SF-based difference in neural processing speed (Breitmeyer, 1975). The trial sequence is displayed in Figure 1C. A fixation cross was presented at the center of the screen (for 500 ms), followed by a blank display (randomized duration, 500–1010 ms). Then, the visual stimulus was presented until a response was made. Participants were instructed to press the 5 key as soon as the visual stimulus was presented. Each participant completed a total of 60 trials: 30 trials for each SF stimulus.

### Results

The proportion of simultaneous responses was calculated for each condition. Two participants were excluded from further analysis, as their SJs were over 25% in the −510 ms or +510 ms SOA condition. To compute the amplitude, PSS, and sigma values, a three-parameter Gaussian function was fitted to each participant’s data based on minimization of the root-mean-square-error (RMSE):

$$P\;\left(\mathrm{response}|\mathrm{SOA}\right)\;=\;\mathrm{Amplitude}\cdot e^{\left[-.5{\left(\frac{\mathrm{SOA}\;-\mathrm{PSS}}{\mathrm{Sigma}}\right)}^{2}\right]}$$

**Figure 2.**
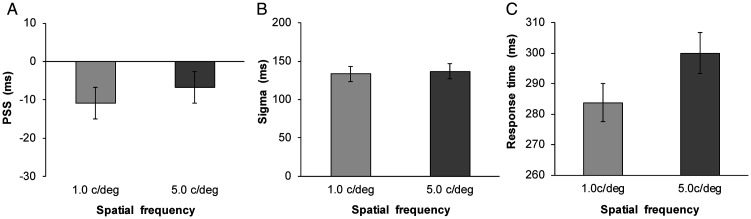
Results of the simultaneity judgment and simple reaction time tasks in Experiment 1. A: Mean estimated point of subjective synchrony. B: Mean estimated sigma. C: Mean response time. Error bars represent standard errors of the mean (*n* = 31).

The SOA parameter was equal to that of the experimental condition (from −510 to +510 ms). The parameters of amplitude, PSS, and sigma values were estimated, and the amplitude and sigma values were restricted to greater than 0. The mean estimated PSS and sigma values are shown in Figure 2A and 2B (1.0 c/deg: mean RMSE = 0.06 ± 0.03; 5.0 c/deg: mean RMSE = 0.06 ± 0.03). The PSS was larger for the 5.0 c/deg visual stimulus than for the 1.0 c/deg visual stimulus, *t*(30) = 2.33, *p* < .05, *d* = 0.20, whereas the sigma did not differ between SFs, *t*(30) = 1.14, *p* = .26, *d* = 0.06. Moreover, the average RT was computed for each SF using data between 200 and 1000 ms (5.05% of the data were excluded). The results are shown in Figure 2C. A two-tailed *t* test on the RT data confirmed that processing speed was slower for the 5.0 c/deg visual stimulus than for the 1.0 c/deg visual stimulus, *t*(30) = 4.88, *p* < .001, *d* = 0.45.

**Figure 3.**
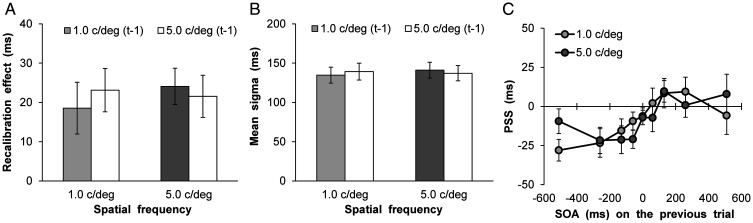
Results of rapid recalibration analyses in Experiment 1. A: Mean recalibration effect. B: Mean simultaneity bandwidth. C: Mean estimated point of subjective synchrony as a function of SOA on trial *t*−1. Error bars represent standard errors of the mean (*n* = 31).

An intertrial analysis was conducted to examine whether the modality order on a given previous trial (*t*−1) affected the distribution of simultaneity responses in the current trial (*t*). Furthermore, the distribution of perceived simultaneity as a function of SOA was compiled for each participant and each SF and trial *t*−1SF separately, given that in some cases trial *t*−1 exhibited either a negative SOA (i.e., audition leads) or positive SOA (i.e., vision leads). The synchrony distributions were then fitted with the above Gaussian function—1.0 c/deg (*t*), 1.0 c/deg (*t*−1), audition leads: mean RMSE = 0.11 ± 0.05; 1.0 c/deg (*t*), 1.0 c/deg (*t*−1), vision leads: mean RMSE = 0.10 ± 0.04; 1.0 c/deg (*t*), 5.0 c/deg (*t*−1), audition leads: mean RMSE = 0.09 ± 0.04; 1.0 c/deg (*t*), 5.0 c/deg (*t*−1), vision leads: mean RMSE = 0.11 ± 0.03; 5.0 c/deg (*t*), 1.0 c/deg (*t*−1), audition leads: mean RMSE = 0.12 ± 0.04; 5.0 c/deg (*t*), 1.0 c/deg (*t*−1), vision leads: mean RMSE = 0.10 ± 0.03; 5.0 c/deg (*t*), 5.0 c/deg (*t*−1), audition leads: Mean RMSE = 0.11 ± 0.06; 5.0 c/deg (*t*), 5.0 c/deg (*t*−1), vision leads: mean RMSE = 0.11 ± 0.04—and the PSS and sigma values were estimated. Moreover, the recalibration effect (PSS when vision leads—PSS when audition leads on trial *t*−1) and simultaneity bandwidth (mean of the sigma values for both audition leads and vision leads on trial *t*−1) were calculated for each SF and the SF on trial *t*−1; the results are shown in Figure 3A and 3B. A two-way analysis of variance (ANOVA) with the SF (2) × the SF on trial *t*−1 (2) was conducted, for the recalibration effect and the simultaneity bandwidth. For the recalibration effect, neither the main effects of SF, *F*(1, 30) = 0.14, *p* = .71, η_p_^2^ = .005, and of SF on trial *t*−1, *F*(1, 30) = 0.05, *p* = .83, η_p_^2^ = .002, nor the interaction, *F*(1, 30) = 0.46, *p* = .50, η_p_^2^ = .02, were significant. In addition, for the simultaneity bandwidth, neither the main effects of the SF, *F*(1, 30) = 0.39, *p* = .54, η_p_^2^ = .01, and of the SF on trial *t*−1, *F*(1, 30) = 0.02, *p* = .90, η_p_^2^ <= .001, nor the interaction, *F*(1, 30) = 2.38, *p* = .13, η_p_^2^ = .07, were significant. In follow-up analyses, I examined whether the PSS varied as a function of the SOA on trial *t*−1. By performing this analysis, we aimed to confirm that the PSS depends on the modality order (i.e., SOAs) on the preceding trial. Figure 3C illustrated how the mean PSS varies as a function of the SOA on trial *t*−1. A two-way ANOVA with the SF (2) × the SOA on trial *t*−1 (9) was conducted. The results revealed a significant main effect of the SOA on trial *t*−1, *F*(8, 240) = 4.58, *p* < .001, η_p_^2^ = .13. Neither the main effect of the SF, *F*(1, 30) = 0.001, *p* = .97, η_p_^2^ <= .001, nor the interaction was significant, *F*(8, 240) = 0.97, *p* = .46, η_p_^2^ = .03.

## Experiment 2

Experiment 1 did not show that differences in SF of the current trial modulate the magnitude of rapid recalibration. Furthermore, the magnitudes of rapid recalibration were almost the same regardless of the difference in SF of visual stimuli between the preceding and current trials. In Experiment 2, I replicated that the difference in SF of visual stimuli did not affect the rapid recalibration. However, Gabor patches of either SFs were repeatedly presented in one session in this experiment. The adaptation to a single SF by repeated presentation may affect the rapid recalibration magnitude. Experiment 2 investigated the effects of repeated presentations of the same SF Gabor patch on the rapid recalibration.

### Method

#### Participants

A total of 32 individuals (18 women and 14 men; mean age = 20.56 ± 2.19 years) participated in this experiment. All participants orally reported normal or corrected-to-normal vision and normal hearing and provided written informed consent before participation.

#### Stimuli

The audiovisual stimuli and experimental conditions were the same as that used in Experiment 1.

#### Procedure

The trial sequence and task were the same as that in Experiment 1. Participants completed two sessions. Each session comprised three blocks; one block consisted of 100 trials and there were 10 trials for each SOA condition. Therefore, each participant completed a total of 600 trials. Unlike Experiment 1, in one session, either of the two Gabor patches was presented. The order of sessions was counterbalanced across participants. After completing the SJ task, each participant performed a simple RT task for each Gabor patch as in Experiment 1.

### Results

**Figure 4.**
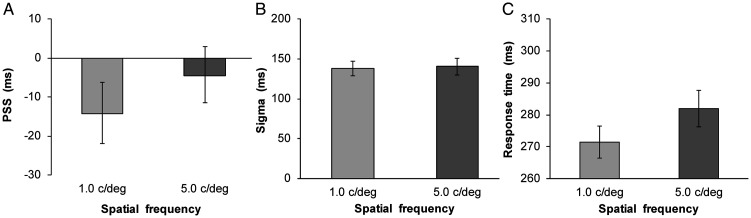
Results of the simultaneity judgment and simple reaction time tasks in Experiment 2. A: Mean estimated point of subjective synchrony. B: Mean estimated sigma. C: Mean response time. Error bars represent standard errors of the mean (*n* = 28).

The proportion of simultaneous responses was calculated for each condition. Four participants were excluded from further analysis, as their SJs were over 25% in the −510 ms or +510 ms SOA condition. To compute the amplitude, PSS, and sigma values, a three-parameter Gaussian function was fitted to each participant’s data as in Experiment 1. The mean estimated PSS and sigma are shown in Figure 4A and 4B (1.0 c/deg: mean RMSE = 0.07 ± 0.03; 5.0 c/deg: mean RMSE = 0.07 ± 0.03). The PSS was larger for the 5.0 c/deg visual stimulus than for the 1.0 c/deg visual stimulus, *t*(27) = 2.83, *p* < .01, *d* = 0.24, whereas the sigma value did not significantly differ between SFs, *t*(27) = 0.53, *p* = .60, *d* = 0.05. Moreover, the average RT was computed for each SF using data between 200 and 1000 ms (3.70% of the data were excluded). The results are shown in Figure 4C. A two-tailed *t* test on the RT data confirmed that processing speed was slower for the 5.0 c/deg visual stimulus than for the 1.0 c/deg visual stimulus, *t*(27) = 3.34, *p* < .01, *d* = 0.37.

**Figure 5.**
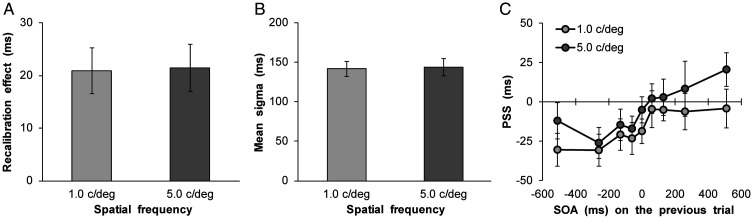
Results of rapid recalibration analyses in Experiment 2. A: Mean recalibration effect. B: Mean simultaneity bandwidth. C: Mean estimated point of subjective synchrony as a function of SOA on trial *t*−1. Error bars represent standard errors of the mean (*n* = 28).

The intertrial analysis conducted was the same as that in Experiment 1. The total distributions were subsequently fitted with the above Gaussian function (1.0 c/deg, audition leads: mean RMSE = 0.08 ± 0.03; 1.0 c/deg, vision leads: mean RMSE = 0.08 ± 0.04; 5.0 c/deg, audition leads: mean RMSE = 0.09 ± 0.04; 5.0 c/deg, vision leads: mean RMSE = 0.09 ± 0.03) and the PSS and sigma values were estimated. Moreover, the recalibration effect and simultaneity bandwidth were calculated for each SF; the results are shown in Figure 5A and 5B. These scores did not differ between SFs for the recalibration effect, *t*(27) = 0.08, *p* = .93, *d* = 0.02, and simultaneity bandwidth, *t*(27) = 0.34, *p* = .73, *d* = 0.04. Finally, Figure 5C illustrates how the mean PSS varies as a function of the SOA on trial *t*−1. A two-way ANOVA with the SF (2) × the SOA on trial *t*−1 (9) was conducted. The results revealed the significant main effect of the SF, *F*(1, 27) = 5.92, *p* < .05, η_p_^2^ = .18, indicating that PSS of 5.0 c/deg was larger than that of 1.0 c/deg. The main effect of the SOA on trial *t*−1 was also significant, *F*(8, 216) = 5.45, *p* < .001, η_p_^2^ = .17. However, the interaction between SF and SOA on trial *t*−1 was not significant, *F*(8, 216) = 0.39, *p* = .93, η_p_^2^ = .01.

## General Discussion

This study examined the effects of neural processing speed based on SF on rapid recalibration. The RT was shorter and PSS was lower for the low SF than for the high SF conditions. These RT and PSS results replicate those of Breitmeyer (1975) and Takeshima and Gyoba (2015), respectively, confirming the different processing speeds for low and high SF visual stimuli. The magnitudes of the rapid recalibration effect did not differ between the low and high SF visual stimuli in both Experiments 1 and 2. In Experiment 2, in which the same SF visual stimuli were repeatedly presented in one block, the estimated PSSs as a function of the SOA on trial *t*−1 was larger for high SF than low SF whereas the estimated PSSs did not differ between SFs in Experiment 1, in which both SFs visual stimuli were randomly presented in one session.

The SF-based differences in processing speed affected the response latency for visual stimuli and synchrony perception for audio-visual stimuli. Low SFs are preferentially processed by transient channels, that transmit information at high speeds, compared with sustained channels, which preferentially respond to high SFs (Breitmeyer & Julesz, 1975; Kulikowski & Tolhurst, 1973; Tolhurst, 1973). Consequently, response latencies are shorter for low SFs than for high SFs (Breitmeyer, 1975). Thus, auditory stimuli needed to be presented earlier for a low SF visual stimulus compared with a high SF visual stimulus to compensate for the difference in transmitting speed (Takeshima & Gyoba, 2015).

However, the magnitudes of rapid recalibration were not modulated by temporal information resulting from neural processing speed of preceding and current trials. The rapid recalibration occurs regardless of the difference in various features (e.g., color, orientation, or location) between adaptation and test trials (Harvey et al., 2014; Ju et al., 2019; van der Burg & Goodbourn, 2015). Similarly, the rapid recalibration was observed when the SF of current trial differed from that of preceding trial in Experiment 1. Moreover, the magnitudes of rapid recalibration were almost same without regard to SF of preceding and test trials in this study. Although physical timing, not the perceived timing, of asynchronous audiovisual stimuli induces rapid recalibration (van der Burg et al., 2013, 2018), the effects of the temporal information resulting in neural processing speed on rapid recalibration have not yet been examined. This study showed that the SF-based differences in neural processing speed were not related to the magnitude of rapid recalibration. Thus, these results also confirmed that intersensory temporal processes are important to producing rapid recalibration, suggested by Harvey et al. (2014).

The present findings, which show the rapid recalibration process is independent of the difference in neural processing speed, would support that rapid recalibration is attributed to changes in the criteria used to judge simultaneity. Yarrow et al. (2011) have suggested that temporal recalibration arises in this manner. An event-related potential study showed that relatively late potentials were modulated by the temporal structure of the previous trial with large SOAs, implying either relatively late perceptual or decision-level audiovisual processes associated with rapid recalibration (Simon et al., 2017). Furthermore, Roseboom (2019) has proposed that shifting PSS according to rapid recalibration is induced by changing the placement of SJ decision criteria in SJ task. In this study, the SF-based difference in neural processing speed, which is temporal information at relatively primal perceptual level, did not modulate the rapid recalibration processes. The present finding also suggests that the SJ criterion changes, which is either relatively late perceptual or decision-level audiovisual processes, underlie the rapid recalibration.

Current results show that PSSs as function of the SOA on trial *t*−1 were smaller for low compared with high SF visual stimuli by repeatedly presenting same SF visual stimuli. In Experiment 2, which same SFs were repeatedly presented in one session, estimated PSS as function of the SOA on trial *t*−1 significantly differ between low and high SFs whereas did not differ in Experiment 1, which both SFs visual stimuli were randomly presented in one session. The PSS score tends to be smaller for low SF compared with high SF visual stimuli in a SJ task (Takeshima & Gyoba, 2015). This difference in PSS would be attributed to the neural processing speed for SFs. Adaptation of temporal information by repeatedly presentation of asynchronous audiovisual stimuli shifts PSS for audiovisual stimuli (e.g., Fujisaki et al., 2004; Vroomen et al., 2004). Temporal information based on neural processing speed might modulate the audiovisual synchrony perception by repeatedly presentation.

This study concludes that the SF-based difference in neural processing speed did not modulate the magnitude of rapid recalibration. Moreover, the difference in SF of visual stimuli between preceding and test trials was not associated with the rapid recalibration process. These findings confirm that intersensory temporal processes are important to producing rapid recalibration. Rapid recalibration could be induced by the SJ criterion changes attributed to the low-level temporal information of audiovisual events.
